# Surplus fat rapidly increases fat oxidation and insulin resistance in lipodystrophic mice

**DOI:** 10.1016/j.molmet.2018.05.006

**Published:** 2018-05-16

**Authors:** Amandine Girousse, Samuel Virtue, Dan Hart, Antonio Vidal-Puig, Peter R. Murgatroyd, Etienne Mouisel, Coralie Sengenès, David B. Savage

**Affiliations:** 1 University of Cambridge Metabolic Research Laboratories, Wellcome Trust-Medical Research Council Institute of Metabolic Science, Cambridge, CB2 0QQ, United Kingdom; 2 NIHR/Wellcome Trust Clinical Research Facility, Addenbrooke's Hospital, Cambridge, UK; 3 Institute of Cardiovascular and Metabolic Diseases, 31432, Toulouse, France; 4 StromaLab, Paul Sabatier Toulouse University, ERL5311, 31432, Toulouse, France

**Keywords:** Lipodystrophy, Energy partitioning, Fatty acid oxidation, Substrate competition, Insulin resistance

## Abstract

**Objective:**

Surplus dietary fat cannot be converted into other macronutrient forms or excreted, so has to be stored or oxidized. Healthy mammals store excess energy in the form of triacylgycerol (TAG) in lipid droplets within adipocytes rather than oxidizing it, and thus ultimately gain weight. The ‘overflow hypothesis’ posits that the capacity to increase the size and number of adipocytes is finite and that when this limit is exceeded, fat accumulates in ectopic sites and leads to metabolic disease.

**Methods:**

Here we studied the energetic and biochemical consequences of short-term (2-day) excess fat ingestion in a lipodystrophic (A-ZIP/F-1) mouse model in which adipose capacity is severely restricted.

**Results:**

In wildtype littermates, this acute exposure to high fat diets resulted in excess energy intake and weight gain without any significant changes in macronutrient oxidation rates, glucose, TAG, or insulin levels. In contrast, hyperphagic lipodystrophic mice failed to gain weight; rather, they significantly increased hepatic steatosis and fat oxidation. This response was associated with a significant increase in hyperglycemia, hyperinsulinemia, glucosuria, hypertriglyceridemia, and worsening insulin tolerance.

**Conclusions:**

These data suggest that when adipose storage reserves are saturated, excess fat intake necessarily increases fat oxidation and induces oxidative substrate competition which exacerbates insulin resistance resolving any residual energy surplus through excretion of glucose.

## Highlights

• In contrast to wild type mice, lipodystrophic Azip mice manifest striking metabolic inflexibility.
• Wild type mice respond to excess dietary fat by storing the surplus energy and thus gain weight.
• In contrast, Azip mice increase fat oxidation and energy expenditure in response to surplus energy intake.
• Unfortunately this apparently ‘helpful’ adaptation also results in substrate competition and worsening insulin resistance.

## 1 Introduction

Securing sufficient energy to sustain life has been the major energetic challenge for all life-forms throughout most of evolution. Usable energy in the form of ATP can be generated by oxidizing any of the major macronutrients, carbohydrate (CHO), fat or protein. Protein, however, is seldom used for this purpose as proteins largely play essential structural and regulatory roles in cellular function. CHO and fat also play important functional roles, particularly in membrane structure and the generation of signaling intermediates in the case of lipids, but in addition act as the major substrates for oxidative energy production.

In contrast to most other species and to their own ancestors, modern humans typically face a very different challenge: the need to manage chronic excess macronutrient intake. Surplus energy can only really be stored as CHO, in the form of glycogen, or as fat, typically in the form of triacylglycerol in lipid droplets, as there is no recognizable storage depot form for protein. Surplus protein and CHO can ultimately be converted to fat for energy storage, whereas excess fat cannot be quantitatively converted to CHO or protein; nor can it be excreted, so it has to be stored or oxidized. In healthy humans, adipocytes have evolved highly efficient mechanisms for taking up and storing surplus fat. In the short term, this response limits exposure of other tissues, which are less well-adapted for lipid storage, to excess lipid thus alleviating the adverse effects of surplus lipid accumulation. In the longer term, the increase in size and number of adipocytes results in a rise in fat mass or obesity.

One of the prevailing hypotheses for the tight association among obesity and its metabolic consequences such as insulin resistance, non-alcoholic fatty liver disease, dyslipidemia, and type 2 diabetes hinges on the notion that the ability to increase the size and number of adipocytes is ultimately ‘limited’ and that, in such circumstances, lipid accumulates in other less well adapted tissues [1], [2], [3], particularly the liver, where it is instrumental in inducing insulin resistance [4], [5]. Terms used to refer to this idea include the ‘lipid overflow hypothesis’ [1], [2], [3] or ‘adipose expandability hypothesis’ [6]. In reality, it seems more likely that in ‘common’ forms of polygenic obesity, the efficiency with which adipose tissue mass increases may well decrease as fat mass expands and similarly the efficiency of postprandial adipose tissue lipid buffering [7] may well decrease, leading to excess lipid delivery to other tissues. This ‘incremental’ change makes it relatively difficult to impose a defined experimental perturbation with measurable outcomes. Lipodystrophy (LD), a state defined by reduced adipose tissue mass and function [8], [9], provides a unique opportunity to directly assess the immediate consequences of surplus fat ingestion in a situation where the capacity of adipose tissue to expand is constrained.

We have previously undertaken an acute, hypercaloric, high fat challenge (30% excess energy as fat) in 7 patients with different forms of partial or generalized lipodystrophy [10]. In that study, we detected a small but significant increase in total energy expenditure, which was almost entirely dependent upon an increase in fat oxidation (29%). However, the study was limited by the diverse range of responses observed in what was a relatively heterogeneous group of patients with different genetic forms of partial or generalized lipodystrophy. We were also unable to ascertain which tissue or tissues were predominantly responsible for the increase in fat oxidation. Here we sought to examine the metabolic and biochemical response to acute high fat feeding in A-ZIP/F-1 (hereafter AZIP) mice, a well-established model of generalized LD [11].

AZIP mice over-express, in an adipose specific manner (by virtue of the aP2 enhancer/promoter), a dominant negative protein termed A-ZIP/F, which prevents the DNA binding of B-ZIP transcription factors of both the CCAAT/enhancer binding protein (C/EBP) and Jun families, both of which are involved in the development and function of adipose tissue [11]. The transgenic AZIP mice are devoid of white adipose tissue throughout life and also have reduced amounts of brown adipose tissue, which expresses very low levels of UCP1 and appears to be inactive [11]. The physiological consequences of the lack of fat are pronounced and include severe liver steatosis, hyperlipidemia and early-onset diabetes [11], [12].

## 2 Methods

### 2.1 Animals

A-ZIP/F-1 mice were initially obtained from the NIH (USA). All A-ZIP/F-1 mice used in this study were hemizygous females on the FVB/N background, produced by breeding hemizygous males with wild-type (WT) females [11]. Female wildtype littermates were used as controls. Four mice were housed per cage, maintained in a 12 h light (06h00-18h00)/dark cycle, and had ad libitum access to a low fat diet (D12450B, Research Diets) or high fat diet (D12451, Research Diets). Animals studied at thermoneutrality were acclimatized to 30 °C for 7–10 days prior to the experiment. For intraperitoneal insulin tolerance tests, an injection of 0.5 U/kg insulin was given to 5 h-fasted (08h00-13h00) mice. Blood collection was performed either on fed or 5 h-fasted animals. Experiments were undertaken on 8–10 week old animals in the animal facilities of the Metabolic Research Laboratories, Institute of Metabolic Science in Cambridge (UK) and of the Rangueil site of US006/Crefre/Inserm/University of Toulouse (France).

### 2.2 Study approval

All animal studies were approved by the UK Home Office and the University of Cambridge according to the Animals (Scientific Procedures) Act 1986 and associated guidelines and/or according to the INSERM Animal Care Facility guidelines and local ethical approval from Toulouse Rangueil Hospital.

### 2.3 Indirect calorimetry

Animals were studied in a Metatrace calorimetry system (Creative Scientific). Airflow rates were 400 ml/min and oxygen – and carbon dioxide concentrations in room air and air leaving each cage were measured every 10 min. Fat and carbohydrate oxidation figures were calculated using the following equations: fat oxidation (mg/h) = [1.695×(VO_2_ l/min × 60)]−[1.701×(VCO_2_ l/min × 60)]; carbohydrate oxidation (mg/h) = [4.585×(VCO_2_ l/min × 60)]−[3.226×(VO_2_ l/min × 60)]. Protein oxidation was assumed to be equivalent to protein intake.

Further methodological details are available in the Supplementary Material.

### 2.4 Statistics

Quantitative data are expressed as mean ± SEM. Multiple group comparisons were determined with 1 or 2-way ANOVA using Graph Pad Prism software. Comparisons between two independent groups were assessed using unpaired Student *t*-tests. Statistical significance was defined as p < 0.05.

## 3 Results and discussion

### 3.1 Baseline characteristics of the AZIP mice

AZIP mice manifest generalized lipodystrophy from birth and so develop severe insulin resistance and diabetes from an early age[11]. We studied female AZIP mice and gender matched wildtype littermates between the ages of 8 and 10 weeks. On a low fat diet (LFD, 10% energy from fat), body weight was similar in both groups but as expected fat mass was substantially lower in the AZIP mice (Table 1). The AZIP mice were also already hyperglycemic, hyperinsulinemic and hypertriglyceridemic at this age (Table 1).

**Table 1:** Body composition and blood biochemistry in female AZIP lipodystrophic mice (n = 8–10) and wild type littermate controls (n = 8–10) whilst being fed a regular low fat chow-(LFD) or high fat diet (HFD). Blood was taken in the fed state for all parameters except for glucose and insulin that were measured in both the fed state and after a 5 h fast.

	Wildtype		AZIP	
	LFD	HFD	LFD	HFD
Body weight (g)	25.9 ± 1.0	27.5 ± 1.1***	26.5 ± 0.8	26.8 ± 0.7
Fat mass (%)	20.9 ± 2.7	29.3 ± 2.2***	5.5 ± 0.8^###^	7.8 ± 0.3^###^*
Glucose (mmol/l)				
fed	10.5 ± 0.3	11.3 ± 0.3	25.6 ± 4.3^###^	34.6 ± 0.7^###^*
fasted	7.9 ± 0.2^&&&^	7.9 ± 1.1^&^	13.4 ± 4.2^##&^	32.5 ± 1.3^###^**^&^
Insulin (μg/l)				
fed	1.1 ± 0.3	1.2 ± 0.2	71.5 ± 21.0^###^	133.1 ± 3.6^###^**
fasted	0.7 ± 0.1^&^	0.8 ± 0.2^&^	16.7 ± 2.7^###&&^	27.6 ± 6.5^###^**^&&&^
TG (mmol/l)	3.8 ± 0.4	2.1 ± 0.1*	14.1 ± 2.8^###^	34.2 ± 5.8^##^**
Urine glucose (mmol/l)	3.2 ± 0.3	4.3 ± 0.2	406.7 ± 19.6^###^	529.6 ± 17.5**^,###^
CHO energy loss (kJ/mouse/day)	0.03 ± 0.00	0.03 ± 0.00	18.10 ± 1.85^###^	18.03 ± 0.36^###^
Liver weight (g)	1.31 ± 0.08	1.35 ± 0.11	3.58 ± 0.15^##^	4.52 ± 0.19^##^
Soleus weight (mg)	n.d.	6.33 ± 0.30	n.d.	5.42 ± 0.73
EDL weight (mg)	n.d.	8.83 ± 0.44	n.d.	8.50 ± 0.31
Gas. Weight (mg)	n.d.	113.26 ± 10.6	n.d.	122.73 ± 4.12

TG, triglyceride; CHO, carbohydrate; EDL, extensor digitorum longus; Gas., gastrocnemius.

*p < 0.05, **p < 0.01, ***p < 0.001 compared to LFD (*t*-test).

#p < 0.05, ##p < 0.01, ###p < 0.001 compared to WT (*t*-test).

^&^p < 0.05, ^&&^p < 0.01, ^&&&^p < 0.001 compared to the fed state (*t*-test).

**Figure 1:**
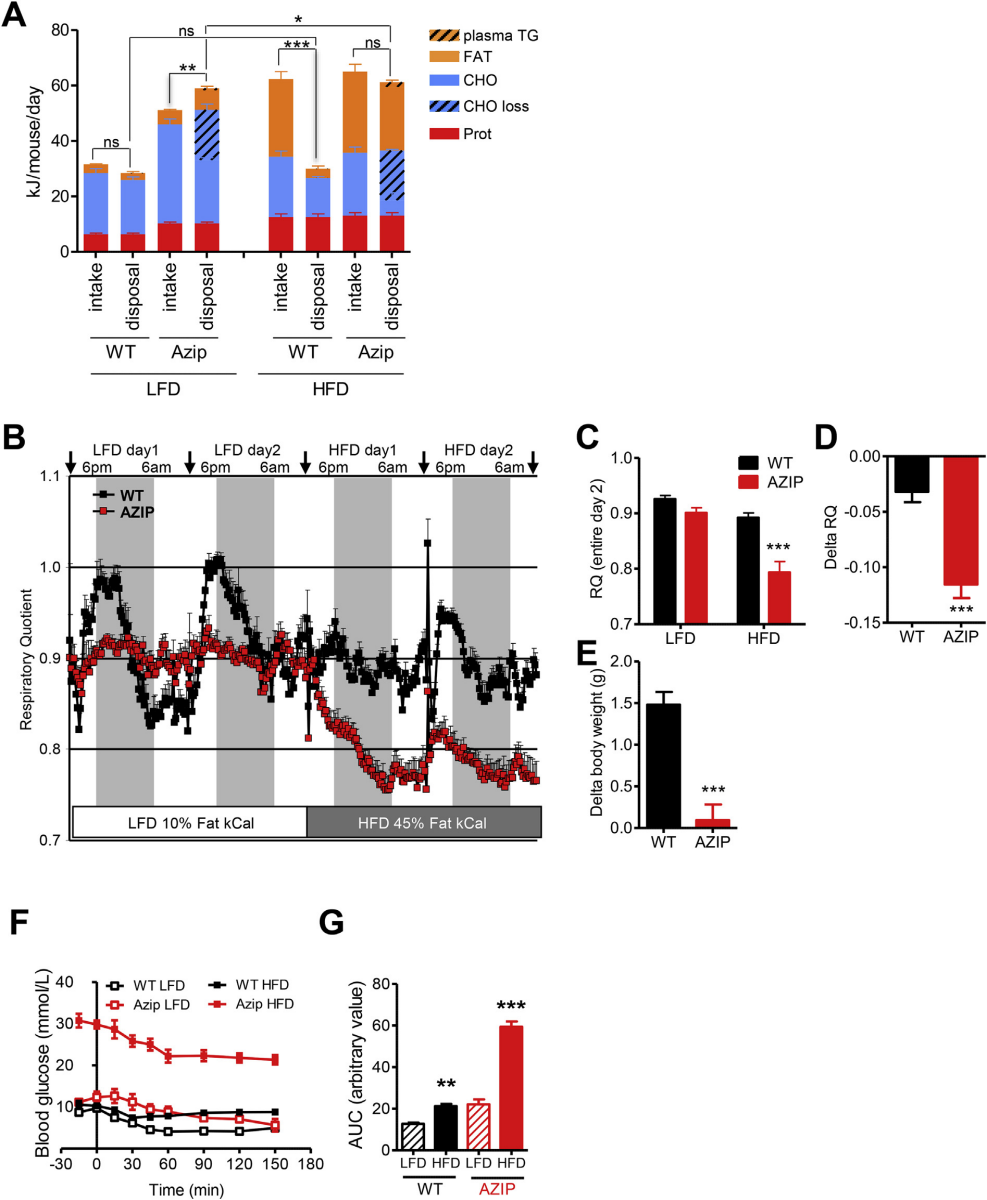
Energetic response to acute high fat feeding in lipodystrophic (AZIP) mice. (A) 24 h macronutrient (protein (Prot), carbohydrate (CHO), fat (Fat)) specific – and total energy intake and disposal in wild type (WT) littermates and AZIP lipodystrophic mice fed either a low fat chow diet (LFD) or after acutely switching from a LFD to a high fat diet (HFD). The estimated (based on measured blood triglycerides (TG) and estimated circulation volume) energy contained in circulating TGs is included in the AZIP data. This measurement is negligible in WT mice. The graph presents the average of the second 24 h period for each diet. (B) Continuous respiratory quotient (RQ) curves assessed by indirect calorimetry in a thermoneutral environment. Arrows represent the daily opening of the calorimetry chambers for animal/food/water weighing. Grey columns represent dark (night-time) periods. (C) Respiratory quotient averages over the second 24 h period. (D) Changes in respiratory quotient, represented as a delta between the LFD and HFD values. (E) Changes in body weight, measured as a delta between the LFD and HFD. (F) Insulin tolerance tests (ITT) performed after a 5 h fast in WT and AZIP mice maintained on a chow (ND) or high fat diet (HFD). Blood glucose concentrations are shown at baseline and following an insulin injection (0.5 U/kg). (G) Area under the curve representation of the data in (F). WT n = 10–14, AZIP n = 8–14; except for ITT data where n = 4–6; AZIP, red bars/squares, WT, black bars/squares. *p < 0.05, **p < 0.01, ***p < 0.001 (2-ways ANOVA and *t*-test).

Indirect calorimetry was undertaken in a thermoneutral environment (30 °C). As expected in a hypoleptinemic lipodystrophic model, total energy intake was significantly greater in the AZIP mice than in their wildtype littermates (Figure 1A) (p = 0.0003). In wildtype mice, total energy expenditure and macronutrient specific oxidation were closely matched to dietary intake (Figure 1A) (p = 0.116). In AZIP mice, energy intake appears to exceed expenditure (p = 0.002) but the apparent discrepancy in energy and CHO balance is accounted for by energy loss in the form of glucosuria (Figure 1, Table 1). Another striking feature of the AZIP mice was the lack of diurnal variation in their respiratory quotient (RQ), which instead largely matched the food quotient of the chow diet (0.940) throughout the 24 h period, reflecting their sustained dietary intake (Figure 1B).

### 3.2 Energy and biochemical responses to excess dietary fat

After the baseline period, both groups of mice were assessed for a further 48 h period during which their diet was switched to a lard-based, high fat formulation (HFD, 45% of energy from fat). This resulted in the expected significant increase in total energy intake in both groups of mice (WT 91% increase, AZIP 27% increase). In the WT littermates, CHO intake remained the same as on chow diet, whereas fat intake increased significantly (∼9-fold) (Figure 1A). Total energy expenditure and macronutrient oxidation rates remained similar to those measured while on a LFD in the WT mice, with the excess dietary fat resulting in a significant increase in fat mass and body weight (Table 1, Figure 1A and E).

In stark contrast to this response, body weight was unchanged in the AZIP group (Table 1), whereas total energy disposal increased slightly (3.76%, p = 0.0355) and fat oxidation rose substantially (226%, p < 0.0001) such that it continued to match fat intake (Figure 1A) (p = 0.083) and resulted in a significant fall in RQ (Figure 1B and C) (p = 0.00013). The relative changes in RQ and body weight are graphically represented in Figure 1D–E.

These energetic changes were accompanied by a significant worsening of hyperglycemia, hyperinsulinemia and hypertriglyceridemia in the AZIP mice (Table 1), the latter accounting for a measurable component of the energy ‘disposal’ (Figure 1B) in these mice. The hyperglycemia, in turn, significantly increased glucosuria (Table 1), which also contributed to excess energy disposal (Figure 1A). The striking changes in fed and fasting hyperglycemia and hyperinsulinemia strongly suggest that the HFD challenge exacerbated the severe insulin resistance present in AZIP mice, but, to directly demonstrate this, we performed insulin tolerance tests in chow and HFD fed mice following a 5 h fast. The short fast led to a more substantial fall in glucose levels in the AZIP mice than in WT mice as expected in mice without any (or very few) functional adipocytes (Table 1 and Figure 1F). During the insulin tolerance test, glucose levels remained significantly higher in HFD fed AZIP mice than in chow fed mice whereas they were similar in chow and HFD fed WT mice (Figure 1F and G). Collectively, these data strongly suggest that insulin resistance worsened acutely following the HFD challenge in the AZIP mice, whereas it was unchanged in the WT littermates.

### 3.3 Tissue specific oxidative responses

**Figure 2:**
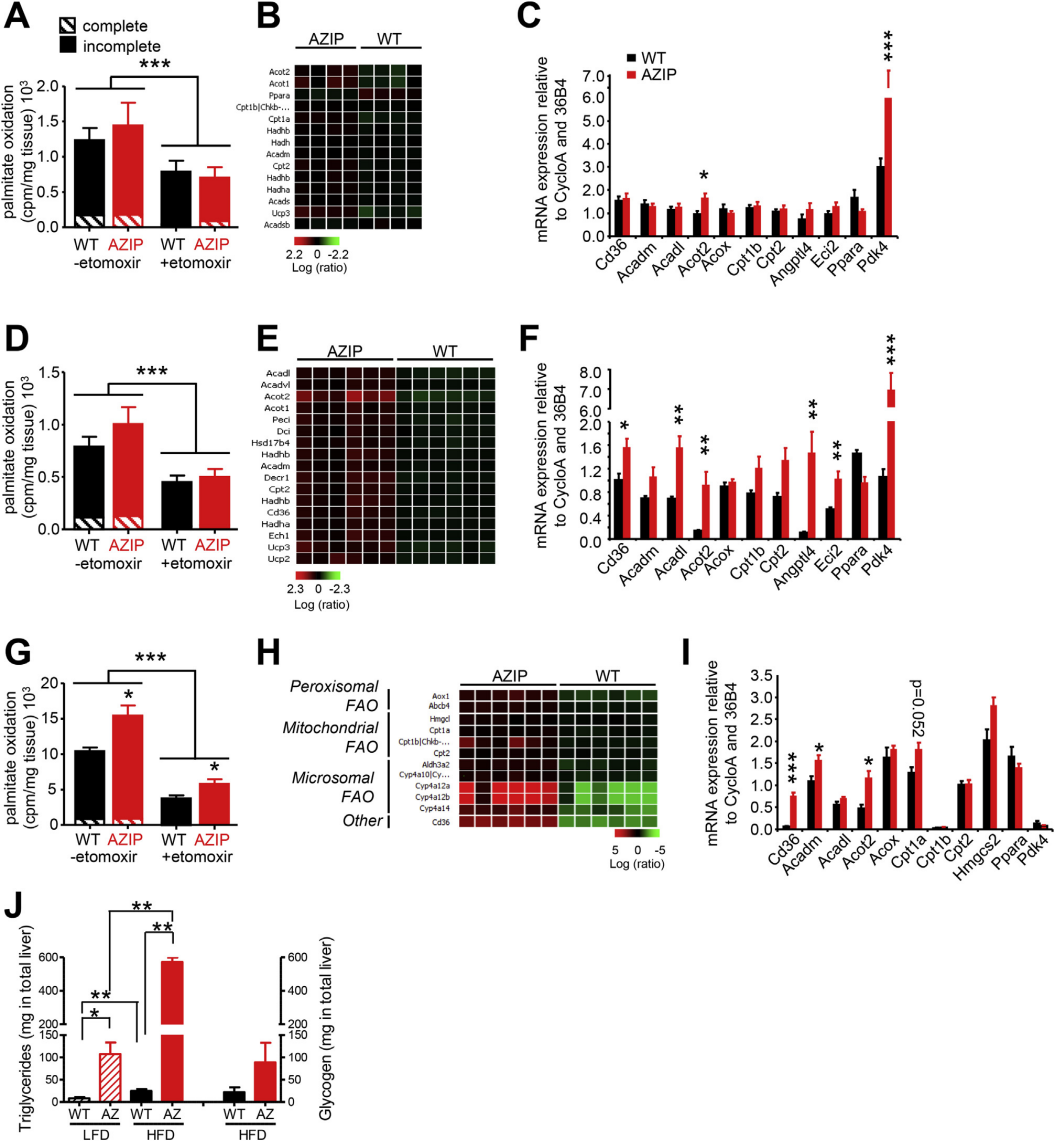
Tissue specific fat disposal in lipodystrophic (AZIP) mice fed with HFD for 48 h. (A-D-G) Ex vivo palmitate oxidation measured in Soleus muscle (A), Extensor digitorum longus (EDL) muscle (D) and liver (G) in the presence or absence of a CPT1 (carnitine palmitoyltransferase 1) inhibitor, Etomoxir. Hatched and solid parts respectively represent complete and incomplete oxidation. (B-E-H) Microarray-based heat maps of mRNA gene expression of Soleus (B), EDL (E) and liver (H) from WT and AZIP mice fed a HFD. The log ratios illustrate relative expression levels of genes involved in the fatty acid oxidation pathway. (C-F-I) Quantitative real-time PCR (qPCR) analysis of genes involved in fatty acid oxidation in the Soleus (C), EDL (F) and liver (I) of WT and AZIP mice fed with a HFD. (J) Triglyceride and glycogen quantification in the liver of WT and Azip animals fed a LFD or HFD for 48 h, measurements done in the fed state. WT, black bars n = 4–6; AZIP, red bars n = 4–6. *p < 0.05, **p < 0.01, ***p < 0.001 (2-ways ANOVA and *t*-test).

AZIP mice lack active brown [11] as well as white fat, so the observed increase in fat oxidation is very unlikely to originate from brown fat. Moreover, the mice were studied in a thermoneutral environment, so were not subject to differential cold-stress, an otherwise common problem for lipodystrophic mouse models housed in conventional mouse facilities. We thus reasoned that skeletal muscle and/or the liver were most likely to account for the increased fat oxidation and so measured tissue specific oxidative rates in ex vivo samples of liver, soleus – (an oxidative muscle type) and extensor digitorum longus (EDL) muscle (a more glycolytic muscle type). Palmitate oxidation rates were similar in both groups of mice in both muscle types (Figure 2A and D) and were appropriately suppressed by etomoxir, a well-characterized CPT1 inhibitor. Nevertheless, we did observe modest increases (particularly in the EDL muscle samples) in mRNA expression levels of several genes involved in lipid uptake, transport and mitochondrial oxidation in AZIP mice compared to wildtype, suggesting that at least EDL muscle may contribute to the overall increase in fat oxidation in lipodystrophic mice (Figure 2B, C, E, F).

In liver homogenates, palmitate oxidation was significantly higher in the AZIP group (Figure 2G), a difference largely accounted for by incomplete fat oxidation. Ingenuity pathway analysis of microarray assessment of hepatic gene expression revealed significant increases in genes linked to peroxisomal-, microsomal- and mitochondrial fat oxidation (Figure 2H). We subsequently confirmed several of these changes using real-time Q-PCR (Figure 2I) showing an increase in the expression of genes involved in the uptake (*Cd36*), processing (*Cpt1b*, *Cpt2*, *Acot2*) and oxidation of fatty acids (*Acadm*, *Acadl*, *Acox*, *Eci2*). Despite the increase in hepatic fat oxidation, liver triglyceride content increased dramatically (∼5-fold) in response to the HFD challenge in the AZIP mice whereas the rise in liver triglyceride was more modest (∼3-fold) in the WT group (Figure 2J). Liver glycogen stores also tended to be higher in HFD fed AZIP mice compared to wildtype littermates (Figure 2J).

## 4 Conclusions

Although often mistakenly perceived as an ‘inert energy storage’ depot, adipose tissue in fact provides an essential buffer for surplus energy intake, shielding other tissues and cell types less well equipped to handle excess lipid. The data presented here provide a graphic demonstration of the metabolic consequences of overloading the energy-buffering capacity, which is dramatically reduced in AZIP mice, of an organism. Since surplus fat which exceeds the storage capacity of adipose tissue cannot be excreted or quantitatively converted into an alternative storage form it has to be stored in ectopic sites such as the liver, remain in the circulation in the form of lipoproteins, or be oxidized. Our data suggest that at least in AZIP mice, the liver is likely to be the major organ responsible for the observed increase in fat oxidation, although muscle, and potentially other tissues such as the intestine, may also contribute to some extent.

The idea that substrate competition might contribute to insulin resistance has long been considered but was arguably most clearly defined by a series of studies conducted by Randle et al. (reviewed in [13]). A key aspect of the Randle hypothesis is that fat and carbohydrate ‘compete’ as oxidative substrates [14]. Randle et al. showed that at least in ex vivo isolated muscle samples, excess fatty acid supplies drove fat oxidation at the expense of CHO oxidation. A series of subsequent studies in animal models and humans have shown that raising circulating fatty acid levels induces fatty acid oxidation and reduces insulin stimulated glucose uptake (reviewed in [15]). This situation is seldom likely to occur in healthy mammals and higher order vertebrates as adipose tissue provides such an efficient ‘sump’ for excess dietary fat [7], favoring weight gain in the face of sustained surplus energy intake. However, in situations such as lipodystrophy, in which fat storage capacity is very limited, our data suggests that surplus fat intake necessitates increased fat oxidation which trumps CHO oxidation, worsens insulin resistance, and leads to excess CHO disposal as glucosuria in diabetics. In many ways, these findings provide a ‘whole organism’ level representation of a key element of Randle's proposal and suggest firstly, that in circumstances where high fat energy intake exceeds the capacity of adipose storage depots, excess fat induces an increase in fat oxidation, and, secondly, that this results in competition between lipid and carbohydrate oxidative substrates and can contribute to insulin resistance.
