# Supplementary material for: Surplus fat rapidly increases fat oxidation and insulin resistance in lipodystrophic mice
Source: Mol Metab. 2018 May 16;13:24–9. doi: 10.1016/j.molmet.2018.05.006 (PMC6026316; doi:10.1016/j.molmet.2018.05.006)
Supplement: Multimedia component 1 [file mmc1.docx]

**Supplementary methods**

*Body composition.* Lean and fat mass were measured in live animals using a Bruker’s Minispec Plus Whole Body Composition Analyser (Bruker). Data are presented as a percentage of total body weight.

*Biochemical analysis.* Fed-state glucose levels were measured in tail vein blood samples with a glucose meter (AlphaTrak, Abboott Animal Health, IL,USA). Plasma insulin, triglycerides and urine glucose were measured by the Core Biochemical Assay Laboratory (Addenbrookes Hospital, Cambridge, UK) with mouse-specific commercial kits [respectively Meso Scale Discovery (Gaithersburg, MD, USA), Siemens Healthcare TG and Siemens Healthcare Glucose (Sudbury, UK)].

For liver triglyceride measurement, a piece of tissue (~100 mg) was homogenized in 1ml of 5% NP40, heated for 5 min at 90°C and cooled down twice, and centrifuged at 12500 rpm for 2 min. Diluted samples were then handled by the ANEXPLO core facility (UMS 006- ANEXPLO/CREFRE, Toulouse, France) for triglyceride measurements. Values were normalized to protein amount in the tissue sample and then adjusted for the total liver weight. The latter is important as AZIP mice manifest significant hepatomegaly (see Table 1).

For liver glycogen measurement, a piece of tissue (~10-20 mg) was dissolved in 1N NaOH for 1h at 55°C and neutralized with 1N HCl before centrifugation 10min at 12000 rpm. Amyloglucosidase (50U/ml) (Sigma Aldrich) was added to the liver homogenate and incubated for 1h at 55°C. After deproteinization, glucose was then measured with the Glucose (GO) assay kit (Sigma Aldrich) according to the manufacturer instructions in the samples treated with or without amyloglucosidase in order to subtract the amount of free glucose. Again, values were normalized to protein amount in the tissue sample and then adjusted for the total liver weight.

*Microarrays.* The transcriptional profiles of samples were probed using the Gene-Chip Mouse Genome 430 2.0 arrays (Affymetrix) as previously described (1). Following normalization, analysis and comparison of transcriptional profiles were performed with the Genespring 11.1 software (2) .

*Analysis of gene expression by quantitative RT-PCR.* Total RNA was isolated from liver, soleus and extensor digitorum longus (EDL) muscles from 4 to 6 animals of each genotype using the RNEasy Mini Kit (Qiagen). RNA (1 μg) was treated with DNase I (Life Technologies) and reverse transcription was performed using the High Capacity cDNA reverse transcription kit (Applied Biosystems). Quantitative real-time PCR was performed on a 7900 HT Fast Real-Time PCR system (Life Technologies) using TaqMan or Sybr Green Master mix. All samples were measured in duplicate and normalized to Cyclophilin A, 36B4 and/or 18s genes. Assay on demand primers were purchase from Applied Biosystems:

| Gene | AOD number |
| --- | --- |
| ACOX1 | Mm00443579_m1* |
| Acadl | Mm 00599660_m1 |
| Acadm | Mm 01323360_g1 |
| Acot2 | Mm 01622461_s1 |
| Angptl4 | Mm 00480431_m1 |
| CD36 | Mm 01135198_m1 |
| CPT1a | Mm00550438_m1* |
| Cpt2 | Mm 00487205_m1 |
| HMGCS2 | Mm00550050_m1* |
| Pdk4 | Mm 01166879_m1 |
| Peci | Mm 0479725_m1 |
| PPARa | Mm00440939_m1* |

*Fatty acid oxidation procedure.* Radiolabeled [1-^14^C]palmitate was purchased from Perkin Elmer (Boston, MA). Palmitate oxidation was measured in liver homogenates and in intact muscles (Soleus and EDL) with a method adapted from Yasdi et *al.* (3). Briefly, tissues were quickly excised and placed in ice-cold homogenisation buffer (250 mM sucrose,10 mM Tris-HCl, 1 mM EGTA, 1% BSA, pH = 7.4). ~50 mg of liver was minced with scissors and further homogenized with a Potter-Elvehjem PTFE pestle in a glass tube in 19 volumes of homogenisation buffer. Liver samples were then diluted 2.5 fold in homogenisation buffer. Liver homogenate (50 μl) was then incubated with 450 μl of the reaction mixture (100 mM sucrose, 10 mM Tris-HCl, 5 mM KH2PO4, 80 mM KCl, 1 mM MgCl2, 2 mM malate, 2 mM ATP, 1 mM DTT, 0.2 mM EDTA, 2 mM L-carnitine, 0.05 mM CoA, 0.2 mM palmitate, 0.3% BSA, 0.125μCi 1-^14^C-palmitate) for 60 min at 30°C. Intact muscles were incubated in 500 μl of the reaction mixture. The reaction was stopped by the addition of 50μl of 70% ice-cold perchloric acid and incubated for 60 min at room temperature. ^14^C-CO_2_ produced during acidification was collected in 100μl of 1M NaOH. ^14^C-labeled CO_2_ corresponding to the complete oxidation of palmitate was determined by liquid scintillation counting. The acid soluble metabolite (ASM) fraction was collected after 16 h at 4°C, and radioactivity was counted. It corresponds to the incomplete oxidation of palmitate.

**Supplementary references:**

1. Kennedy, A.R., Pissios, P., Otu, H., Roberson, R., Xue, B., Asakura, K., Furukawa, N., Marino, F.E., Liu, F.F., Kahn, B.B., et al. 2007. A high-fat, ketogenic diet induces a unique metabolic state in mice. *Am J Physiol Endocrinol Metab* 292:E1724-1739.

2. Badman, M.K., Pissios, P., Kennedy, A.R., Koukos, G., Flier, J.S., and Maratos-Flier, E. 2007. Hepatic fibroblast growth factor 21 is regulated by PPARalpha and is a key mediator of hepatic lipid metabolism in ketotic states. *Cell Metab* 5:426-437.

3. Yazdi, M., Ahnmark, A., William-Olsson, L., Snaith, M., Turner, N., Osla, F., Wedin, M., Asztely, A.K., Elmgren, A., Bohlooly, Y.M., et al. 2008. The role of mitochondrial glycerol-3-phosphate acyltransferase-1 in regulating lipid and glucose homeostasis in high-fat diet fed mice. *Biochem Biophys Res Commun* 369:1065-1070.
